# Supplementary material for: CRISPR-Cas13d screens identify KILR, a breast cancer risk-associated lncRNA that regulates DNA replication and repair
Source: Mol Cancer. 2024 May 15;23:101. doi: 10.1186/s12943-024-02021-y (PMC11094906; doi:10.1186/s12943-024-02021-y)
Supplement: Supplementary file 1 — Supplementary Material 1 [file 12943_2024_2021_MOESM1_ESM.pdf]

Supplemental File 1. KILR sequence (5' to 3')

TTTTTTTTTTTTTTTTTTTTAGATCTTGCCTCCAAAGTAGAGCTTGGGGACTGTTGTCTAATCCAAACCACCAGG
GGAAATGCCAGTTGTGTGGGCTGTGACTTTGGGATGAGACCGCAGAGCCTGTAGAATCAAGTTGAACCTCAAAGT
GCCAGCCTTGTGGATTTCGGCCCACAGGCTGCTCCAGCAGAATGTTCATGCAGATGCTCTTTGAACAAACTCCCT
CCTCAAACTGCAAATTCAGCCTTCCCAAATGGATTTGTCAAGTTGGAAATCTCTTATATATACCCATCCTCACCC
TTCGTAGGCAGAAGCAATTCTAGAGCTGTCTTCTCAGCGAATTGTTAACCAACTCAGAGCCTGGTGGTGTACCGG
GAGTGGCAAGTTCTGTCAGTTTATTTCAGGACAGCCAGAAGGTGTTCGGTGGAACTGGTCCCGTGCCTCTGCAGT
GCAGGTGTGGGCCTGGTGGGGTGGGAAGTGCAGCCAGGGAAAACACAGGGCAGAAGCCTGGGCAGAAACTGTCCT
TATGGAATCTGACTTTCCTACTCCCTACCCAGGTCACAGATACAGTGCTAGGATCCTTGGACAGACCATGAAACC
TGGAGAATTGGAATTTCAAGAAGAAAATGGTTTAAAATGCCATAATCTTGCATTGAATTAATTGAAGAGGAAACA
ATATTTGATTACAGCTATCTGATACCACTTCAAAATGGCAAGCCATTAGATTCAACTCAGTTCAACAAACTGGTC
CCTACTGTGTGCTAGGGTCTGTGTTAGGCACTGAACATAATGCCTGGATACAATGAATCCCATAGGGTTCAGCTT
CTACCAAGACAAGAAAGTTATCTTGGGGGGGGAAAAAAAAGTAAAAAGTACTTGTCAAAGAAACACATTTTTCCT
AAACAAAACTTAACATCATAAATCTGGAGAGAAAAATATCCAGTAATAATATACTCTCTGAATGGAAAATTCCCA
CAATAATAACAAAAATAACAATGATCTGTATACTTAGTACAGCTCTATTTTTGCTTTTCATCTAATGATTTCAAA
GAGTTTAGCAACCACTGCTGAGCTAATCCTTAGTACTAAACTTGACTCTTCCATCCTTCTTTGAAAAAAAAAAAA
AAATTGGCACTAACTCCTCAACATCCCACAGGGAAGAAGTTCTGGAAACAAAGCATGGCTCACTCCCACACAAGC
ATCAAGCAGCAAATTAAAGACAGTCTAATTCATCCAGAAAAGTAAGTTGGGACCCAGAAGAATCAAATGAATGCC
CACAAAATGAAAGTCATTTGAAAAAGAAAGACAAGTTTCTTTCTTGCAGGTGAAAAGGAAAAACTTTTCAATGTA
CCATTGGGCATTCTTGATGGGTTTATATCTCTCAGAGATGTAAGTTCCAGAGTATACTCTGGGAAGGCTTTCATT
TTCAAAGACAGAATTTAAATCCTGTTTTAAAAACAGAAAGGGAGATAAAGATTTCATTTCCATAACATGGTATGT
AAAGACAACTTGATTCAGCTGATGATCTCCAAGCTCTTTGATGAATCCCCTCAGAACCACATCCATGTAAAGGGA
AAGGTTATGAATGAAAGAATTCAATTGTTTGCAAAAATAAAGGGAGACTCTGCCAGAGATAGAGATGATCTGGAA
CAGAACTGGTCCCTAATTACACAGTAATCTGACACCTTAGCACTCTCATCCTGCAGTCTTCACCTTCAAAGGATC
TTCCCAATATTAATGAATTCTTCACCTCCTTTTCTCTCATCTTATCCATAATAGCTACTCTTTAAAGAAAAGTTG
GTCAACTTTTTAGAAGACTTGACTAACATTATCATTTTAGCCTAGGATTTAGTCACAAGTATCTCTTTCCTTTTG
ATGCTTTGTGGTGTTTCCTTTTGCCTGGTTTTGAATACATATTGCAGGCTGTGTTTATGACCCTTTGATTTTTTT
TTTTTTTTCTTGAGACAGAGTTTGGTTCTTGTTGCCCAGGCTGGAGTGCAATGGCACAGTCTCAGCTCACTAAAA
CCTCTGCCTCCCGGGTTCAAGCGATTCTCCTGCCTCGGCCTCCCGAATAGCTGGAATTACAGGCGTGCATCACCA
TGCCCGAATAATTTTGTATTTTTAGTGGAGACAGGGTTTCACCATGTCGATCAGGCTGGTCTTGAACTCCTGACC
TCAGGTGATCCACTCACCTCAGCCTCCCAAAATGCTGGGATTACAGGCATGAGTCACTGTGCCTGGCCTGAAACT
GTTATTACACACACACATACACACACGGCAGCCTTGCTGGGAAGATATTATAATAAAATAATATAGAAGTATTAC
CATAGGACAAAGATCAGAGATGGCTTTCTAAAAGCCTCAAAGACATGTGTGAGCTGGGTCCTAACAGATAACTAG
GAAGGTGTCAAGTGTGTAAGAGGGAAAGGCATCCTAGGCAGAGGGGAGCTGTTTGGAGAAAGGCTGGTGACATGG
ATGGGAATTGATGGTCCAGGTATAAGGCACACTGAGGTGCAGCTAGAGCCCAGGCCTGGGGTATATATTGTGTTA
ATCAGAATTTGACTGGAAAAGTGACTTGCTCAAGCTTACACAGTCAGAAAGTAGCACTGCTTGATCTCAGTGAGG
CTATCTGGCTCCAAGTCCATGCTCAGTCTGAGTTAGCATCCCAGTCCTTCTCCTTGTGCAAAAAATGAATGGATA
CACGGAGCTTACAGCCAAATGGGTGATACAGGCATGTAAATTCACAATTACAATACATGCAAGAAACATCTGTCA
AATACCTTCTACGTGCCAGTAGCTATGCTAGGCAATGCGAATACAAAAAAAAAAAAGCCCCTCTCTTTGAATGAA
GCCTAAGGAGTCTGGTTAAGAAGAAAAATAGCAGTTACAATACCTGTCCAAAGTGCTGGGGAACCAAAGGAGAAA
TCTGCCATACAGAGTGAGAGAAGGTGCCTCAGAGGACTTGAGCTAAGACTGGAAGACTCACAATTCAGTGGTTGG
CACTACCAAGGCAAGATCATGATCATTGCTAACATGAATAAGCACTTAAGAGTATGCCAGGCACTCTTCTAAGCT
CTTTATCCATATTAACTCATTTAGTCCTCACAACAATCCCCTATTCTACAAACAAAAAGGTGAAAGATTTTGCCC
AAGATCACACAACACATAAGTGGCAGTGCCAGAATTTGAATCCAAGTTGTTTGGTGCCAGAGCCCATACACTCGC
CACTATACTACATTCTCTTGCTTTCACAGAGAGGGAAGCCCAGGCCTGCTGAAGTCAGGGAATGTTTCCAAGAGG
AGGTAACATGTGAAATCATTCCAAAAAGATCTCTGGGAGTTGAGTTGTTGAGCCAGGAGACGAAGAGTATGGCCA
AGGGCATAAGAGTCAAGGTAGGATGAGAGACAAGTGTTTTCAAGGAAGAGCAATTACTTCCTGGTAGCTGGAGGG
TGAGGCTGGTTTTGGAGAATGGTGGACCCCTGTAAAGGAGTAGGCAGGGAGTACGTTATAGTTTGGTTTGTTGGT

TTTCAGGGAAGACAAACTTATACCAATAGTCTAAGTTAAAGGATACAAAGGACCAGGGATCAGAGACTAAACTCA
GCCTGGACACGTTTGCAGGCTGCCCACTCTCCTGTGGGTGGTGCTGCCTCCAGTTTCACCTTCTACTTTGTTTGC
TGCATCACTGAACAGCAGAAGTGGCCTGAATTAGTGTGATGCCATTGGAACTGGAGAGCATGGGCTGAATGGAAT
GGATTTGACATAAAGGAGAGGAAGCAGGGAAGGGGACTACAGAGTTCCTAGCTCGGGTGCATTGCAGAGATGGGA
AATAAAGCAGGAGGAGCAGATTTGGATGGTGTTGCGGACGGAACTGTGTTCCTCCCAAATTCATATGCTGAAGCG
CTAACCCTGCAGTGTGACTCTATTTGGAGATAGGGTTGTTAAAGAGGTAATTAAGGTTAAACAAGGGCATAAGGG
TGGGCCCTAATCTGGTAGAACTGATGTGTTCATAAGAAGAGAGAGATGCCAGAGAGCCCTCTCTCTCCAGGCTCT
CACACAGAGAAGAGGTCATCTACAACCAGGAAAAGAGGCCTCAACAGAAACCAACCCTGCTGACACCTTAATCTT
GGACTTCCAGCCTCCAGAACTGTGAGAAAATTAATTTCTGTTGTTTAAGCCACCCAGTCTATAGTATTTTGTTAA
TAGTACCCCAAGCCGATTAATACAAAAGAAAAGACTATTATCAGCCTTTGGATGAAGACATAATAGATGGATTTC
AAAACAAATCTTGAGGCCGGGTGCAGTGGCGCATGCCTGTAATCCCAACACCTTGGGAGGCTGAGGTGGGAATAT
CACTTAAGCCCAGGAGTTCAAGACCAGCCCAGGCAACATAGGGAGAACCCCCCGCCCCCACCCCCCCACCCCCCA
CCGGGCTTCACACACAAAAAATAAAAATTAGCCGAGCATGGTGATGTGTGCCCGTAATTCCAGCTGCTTGGGAGA
CTGAGGTAGGAAAGTCACTTGAGCCTGGGAGGTCAAGGCTGCAGTGAGCTGAGATGGCACCACTGGAGGCCAGCC
TGGGTAGCCCTATCTCAAAAACAAACAAACAAATCTTGAGACACAATTGGCAGGGCTTGGTGCTTCAGTGGAAGT
GGAGCCTGAGAAAGAGTGAGAAGGAGGATGGGATATTGTCCAGGTTTCAGTTATGGATACAGGGTGAATTGTGGT
GCCTTTCCCTGAGTCAGAGATTTGGGAGGTGCACCTGGGCGGAGGGCGAAGGCATAGTTAGGATTTCTTGGTTGT
AAGCAAGATAAATTTAAGACAGGGCTGATTAACTATAGGGATGTAGTGAGACTAAAAGGGAAGTGGAACCTCATT
CAAACCCAACATCAGCACTCTAGGAGAGTGAGACTCCTTGGAGGCCAGACTAGACGGTGGGTCTGGATACAGAGC
AGCCCCAGGCCCTCAGCAGGTGCTCACGCCAGCACGGGACTGGGCTGATTCCATGGCCCTGCCCCCTTTCTTCTA
ACTACTGACTACTCTGACCCTGATTCCTAGAGAAAGAGAGACTGTTTGGCTCAGGAGTTTCAATCCAGCCTTCTA
GTTAGCCCAGGGATTAGCTGCCCAGGGACCCATCCTTGGGCACATCCCTTATAGTTGCTGGTCATGTGACAGACC
ACTTGGCTTGACAAATCTGTCCCTTCATCAGTGGCTGTGAGCAAGTCAGCATCCTTAGAAAGGTCTGAGGTATGT
CAGGCACTGTAATAGAAACACCTAGAACAGAAGGTGGGCGTGGTAAGAAGACCATTGTGTTGGTTTTAGGCTTAT
TGGGTTTGGAGGATCTGCTTGACATGCAAGCAGAGAGACCCAGAACATATATCTACAATAGGCACCTGGAGACAT
GGGCCACTCAACAGAGCAATGAAGGCCATGGAGACAGATTTCAGAGATGACAGCACATGGACGGACCCAATTCCT
ATTAAATAAGCAATAAAGGGACACATACTCATGTTGCAAAATTCAAACAACACCAAAGCATGGAGAATAAGAAGT
TAAAGTTCCTCAATGCTACTCTCTTCTCCCAAAGTCACCACTGTAAATGATTTTATGTGTTTTCTTTTTACTCCA
CTGCAGTTACATTTGTAAAATATGTATGTATCATTTTAGGTCAGAGTTTTTTTTTTTCAGCAAATGGGCTTATAC
TTTTCATTAATTTTTTTACTTGCTTAATAAAATGTCTTGGAGACCACTGCACTCAGCACTTACAAACCTACATTA
TTCTTTATATCTATAGATAGTGTTCTGTTGAATAGATGAGATATAATTTATTTATTCACCATCTGCCTCTGAAGT
TAGATTGTATCCAATGATTCTTTATTATAAACAATGCTTCAAGAGCACCCTTGTACATATATCTTTGTGTGCAGC
CCATTTCTAGAAATGATTTACTGCGTTGAAAGGATGTGCATTTTCCCACTGTCCTCTAAAAAGACTGTACCATT
TACCCTTCTAGCAATGATAGAGGATGCACATGCTTATTGCTCTACACCCACGTGGTTGTTTCTTAAACCATTTTG
CATCAGACTCCTTTAAGATGTTGAAAGCTATGGATTTTAATTCTAGAAAAATACACAGATGTACATATACAGAAC
ATTTTGCATACAATTTCTGCAGGTTCACAGACAACCTAAACACACCCCAGAACACACCCTTCCCCACCCCCTGCC
AGACCTGAATAGACATGTGTGAATGGGTGCCAGTCTAAGGGCAGTGGGTCCCAATCTTAGTTGCACATTAGAATC
ACCTGCAGGACTTTGAAAAGTCCCCATGCCCAGGCCACTTCCCAGACCTATCGATCTGGTTCTTTTGGAAAAGGA
TCTGGGTATCAGTATTTTTTTGCAAGTTCTCCAGGTGATATTAACATGCAGCCAATCGCAGGAACCACTGCAGTCT
AGGAGAGGCCTGAAGAAGGATTAAAAAGAATTTAATAACAGAAAACCAGAGTGTACAAGGGGCTCTTGGGGGGCT
CACACGTGGCTGGAGGAGCTTTTATTTTAACAAATCTGGGGTGGGATCTGGCATTGATACATATTTTTTGTTGTG
AGAATGTTTGTCATACACAAAAGTAGAGAGAATGAACACCCATGTAACCTATATTAAATACTTTAAAAAAA
