# Supplementary material for: CRISPR-Cas13d screens identify KILR, a breast cancer risk-associated lncRNA that regulates DNA replication and repair
Source: Mol Cancer. 2024 May 15;23:101. doi: 10.1186/s12943-024-02021-y (PMC11094906; doi:10.1186/s12943-024-02021-y)
Supplement: Supplementary file 6 — Supplementary Material 6 [file 12943_2024_2021_MOESM6_ESM.pdf]

a

K5+/K19+	Day 0 - Rep 1	Day 0 - Rep 2	Day 0 - Rep 2	Day 21 - Rep 1	Day 21 - Rep 2	Day 21 - Rep 3
Day 0 - Rep 1	NA	0.987	0.987	0.927	0.923	0.927
Day 0 - Rep 2	0.987	NA	0.985	0.926	0.925	0.925
Day 0 - Rep 2	0.987	0.985	NA	0.927	0.924	0.927
Day 21 - Rep 1	0.927	0.926	0.927	NA	0.957	0.960
Day 21 - Rep 2	0.923	0.925	0.924	0.957	NA	0.924
Day 21 - Rep 3	0.927	0.925	0.927	0.960	0.924	NA

MCF7	Day 0 - Rep 1	Day 0 - Rep 2	Day 0 - Rep 2	Day 21 - Rep 1	Day 21 - Rep 2	Day 21 - Rep 3
Day 0 - Rep 1	NA	0.992	0.991	0.908	0.892	0.901
Day 0 - Rep 2	0.992	NA	0.990	0.906	0.891	0.899
Day 0 - Rep 2	0.991	0.99	NA	0.908	0.892	0.901
Day 21 - Rep 1	0.908	0.906	0.908	NA	0.932	0.945
Day 21 - Rep 2	0.892	0.891	0.892	0.932	NA	0.923
Day 21 - Rep 3	0.901	0.899	0.901	0.945	0.923	NA

MDAMB231	Day 0 - Rep 1	Day 0 - Rep 2	Day 0 - Rep 2	Day 21 - Rep 1	Day 21 - Rep 2	Day 21 - Rep 3
Day 0 - Rep 1	NA	0.984	0.985	0.916	0.906	0.911
Day 0 - Rep 2	0.984	NA	0.984	0.916	0.910	0.912
Day 0 - Rep 2	0.985	0.984	NA	0.915	0.906	0.912
Day 21 - Rep 1	0.916	0.916	0.915	NA	0.941	0.946
Day 21 - Rep 2	0.906	0.910	0.906	0.941	NA	0.941
Day 21 - Rep 3	0.911	0.912	0.912	0.946	0.941	NA

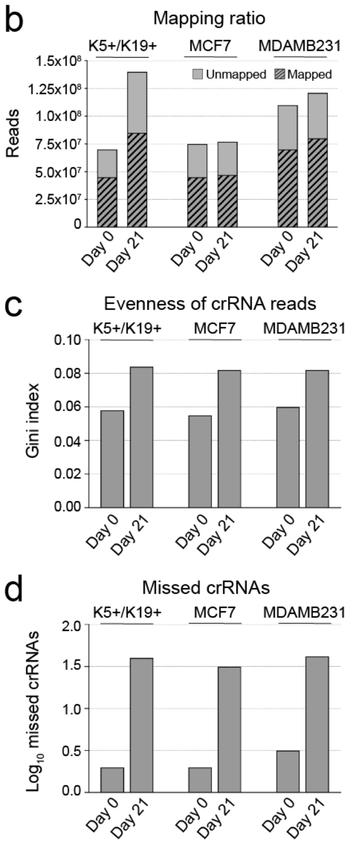

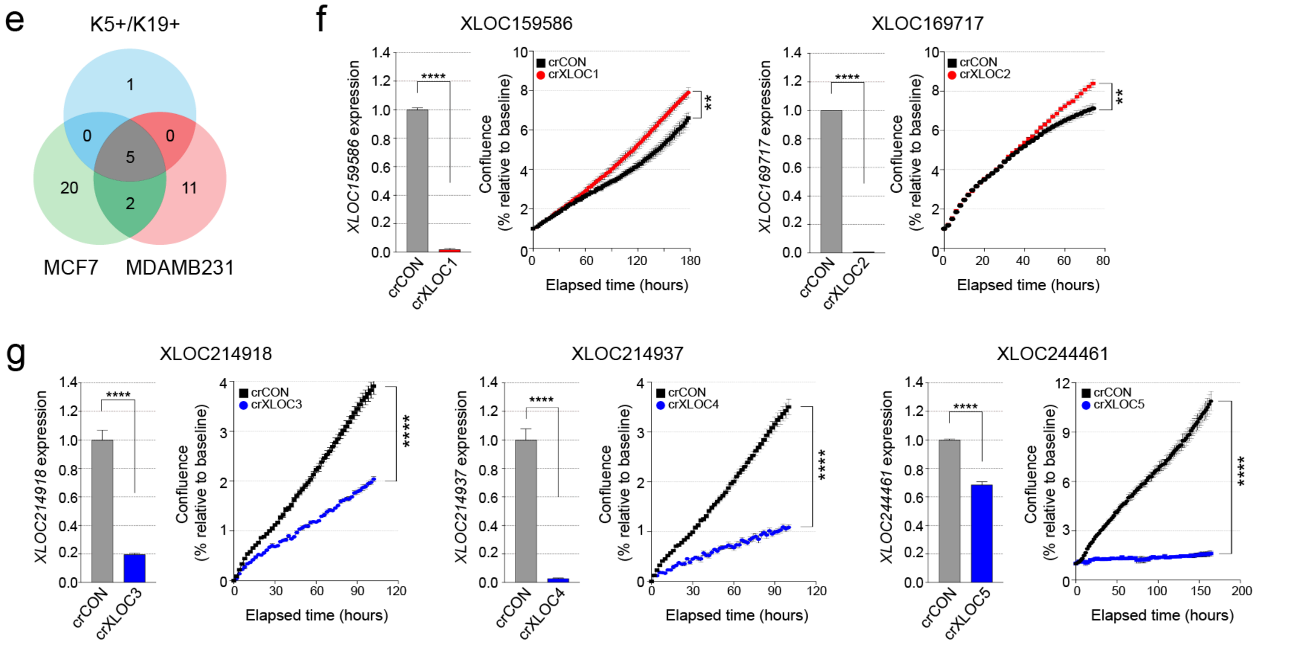

Supplementary Figure 1. Quality control assessment of the CRISPR-Cas13d screen data. (**a**) Heatmaps showing pairwise sample correlations between CRISPR-Ca13d biological replicates in K5+/K19+, MCF7 and MDAMB231 breast cell lines. (**b-d**) Graphs generated by performing MAGeCK crRNA counts in each cell line at Day 0 (n = 3) and Day 21 (n = 3). (**b**) The total number of reads and mapped percentage of crRNAs. (**c**) Gini index measures the evenness of crRNA read depth. (**d**) The number of missed crRNAs. (**e**) Venn diagram of high confidence lncRNA hit counts with FDR ≤ 0.3 in three breast cell lines. (**f, g**) qPCR for lncRNA expression (left panels) and cell confluence measured over time using Incucyte (right panels) in MCF7 cells after CRISPR-Cas13d lncRNA knockdown. The crCON contains a non-targeting control. Error bars, SEM (n = 3). p values were determined by Student's *t*-test (**p < 0.01, ****p < 0.0001).

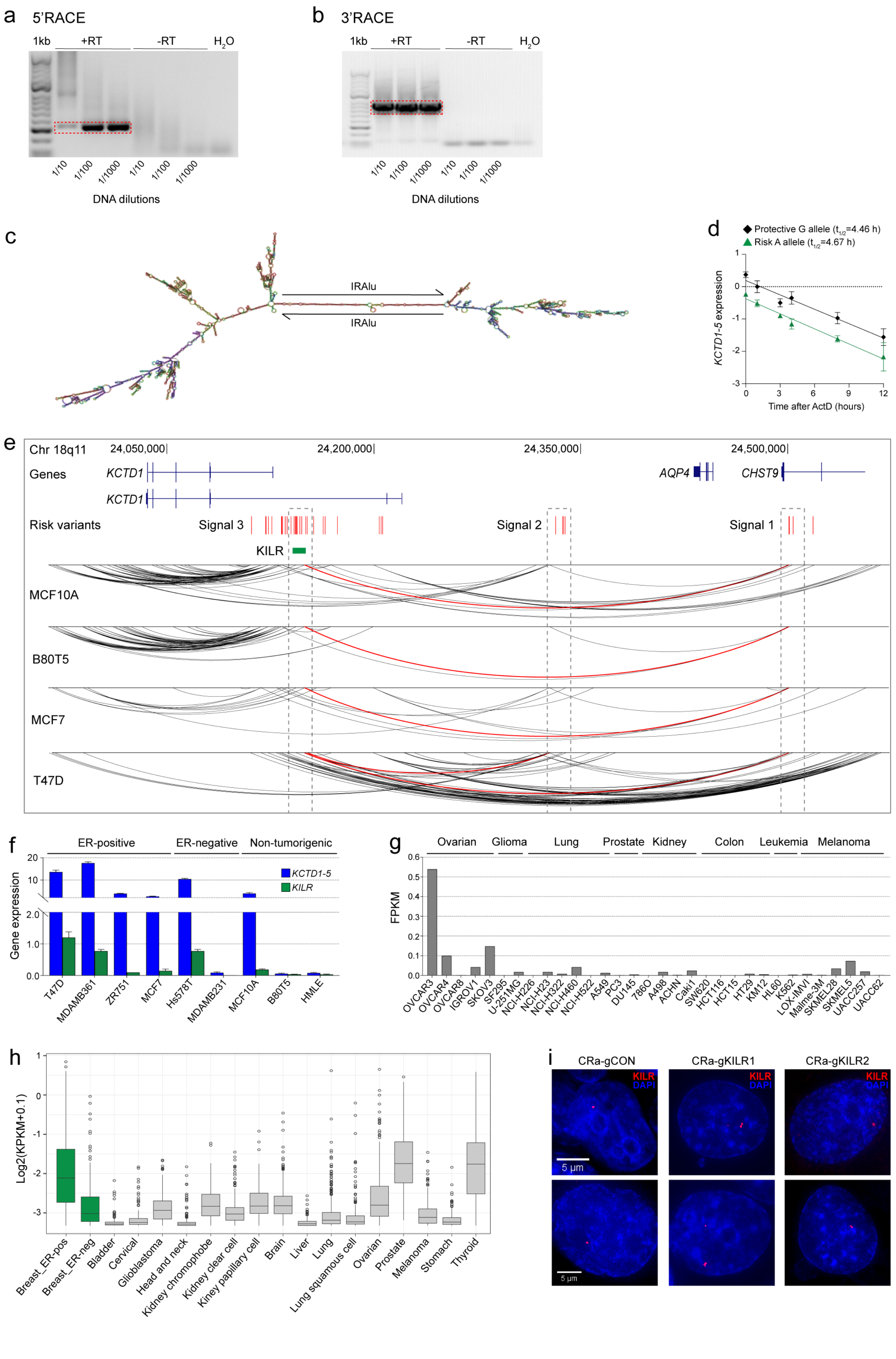
a
5'RACE
1kb
+RT
-RT
H2O
1/10
1/100
1/1000
1/10
1/100
1/1000
DNA dilutions
b
3'RACE
1kb
+RT
-RT
H2O
1/10
1/100
1/1000
1/10
1/100
1/1000
DNA dilutions
c
IRAlu
IRAlu
d
Protective G allele (t1/2=4.46 h)
Risk A allele (t1/2=4.67 h)
KCTD1-5 expression
Time after ActD (hours)
e
Chr 18q11
24,050,000
24,200,000
24,350,000
24,500,000
Genes
KCTD1
KCTD1
AQP4
CHST9
Risk variants
Signal 3
Signal 2
Signal 1
KILR
MCF10A
B80T5
MCF7
T47D
f
ER-positive
ER-negative
Non-tumorigenic
Gene expression
KCTD1-5
KILR
T47D
MDAMB361
ZR751
MCF7
Hs578T
MDAMB231
MCF10A
B80T5
HMLE
g
Ovarian
Glioma
Lung
Prostate
Kidney
Colon
Leukemia
Melanoma
FPKM
OVCAR3
OVCAR4
OVCAR8
IGROV1
SKOV3
SF295
U-251MG
NCI-H226
NCI-H23
NCI-H322
NCI-H460
NCI-H522
A549
PC3
DU145
786O
A498
ACHN
Caki1
SW620
HCT116
HCT15
HT29
KM12
HL60
K562
LOX-IMVI
Malme-3M
SKMEL28
SKMEL5
UACC257
UACC62
h
Log2(KPKM+0.1)
Breast_ER-pos
Breast_ER-neg
Bladder
Cervical
Glioblastoma
Head and neck
Kidney chromophobe
Kidney clear cell
Kiney papillary cell
Brain
Liver
Lung
Lung squamous cell
Ovarian
Prostate
Melanoma
Stomach
Thyroid
i
CRa-gCON
CRa-gKILR1
CRa-gKILR2
KILR
DAPI
5 µm

Supplementary Figure 2. *KILR* is a large, single exon, breast cancer-associated lncRNA. (**a, b**) Agarose gels of 5'RACE (**a**) and 3'RACE (**b**) PCR products. The dashed red outlines highlight the *KILR* transcript. RT (reverse transcriptase). (**c**) The predicted secondary structure of *KILR* (RNAfold). The black arrows show the inverted repeat Alu (IRAlu) elements predicted by Dfam [1]. (**d**) *KCTD1-5* hnRNA stability assay in MDAMB361 cells after treatment with actinomycin D (ActD), then qPCR for *KCTD1-5* hnRNA relative to *CDKN2A* mRNA levels. *KCTD1-5* hnRNA half-life ($t_{1/2}$) was calculated by linear regression analysis. Error bars, SEM (n = 3). (**e**) WashU genome browser (hg19) showing annotated GENCODE genes (blue) and *KILR* (green). The breast cancer risk variants are shown as red vertical lines (Signals 1-3). Variant capture Hi-C chromatin interactions in breast cell lines are shown as arcs. The dashed gray outlines and red arcs highlight chromatin interactions between risk variants and *KILR* in MCF10A, B80T5, MCF7 and T47D cell lines. (**f**) qPCR for *KCTD1-5* and *KILR* expression in ER-positive, ER-negative breast cancer and non-tumorigenic breast cell lines. Error bars, SEM (n = 3). (**g**) Normalized FPKM expression of *KILR* (estimated by LocExpress [2]) from AnnoLnc2 [3] in the Cancer Cell Line Encyclopedia. (**h**) Boxplot of FPKM expression of *KILR* in The Atlas of Non-coding RNA in Cancer (TANRIC [4]) from TCGA RNA-seq data (6,000 tumor samples from 17 cancers). (**i**) Additional confocal microscopy images of *KILR* in MCF7 cells after CRISPRa activation of the *KCTD1-5* promoter to overexpress *KILR* with two independent gRNAs (CRa-gKILR1-2) stained with Stellaris *KILR* RNA FISH probes (red). The CRa-gCON contains a non-targeting control. Nuclei were stained with DAPI (blue). Scale bar, 5 μm.

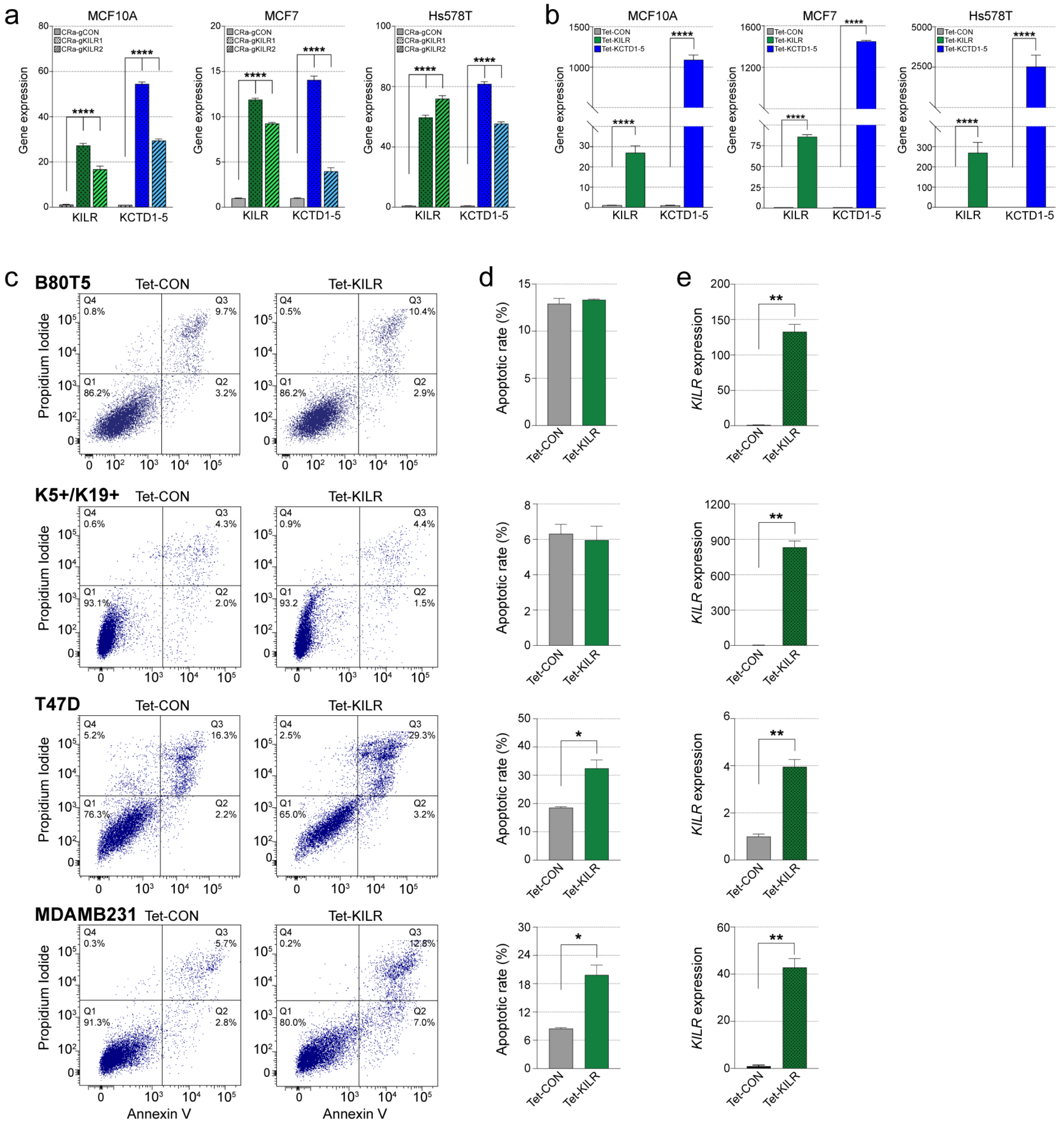

Supplementary Figure 3. *KILR* overexpression induces apoptosis of breast cancer cells. (**a**) qPCR for *KILR* or *KCTD1-5* expression in breast cells after CRISPRa activation of the *KCTD1-5* promoter to overexpress *KILR* with two independent gRNAs (CRa-gKILR1-2). The CRa-gCON contains a non-targeting control. Error bars, SEM (n = 3). p values were determined by two-way ANOVA followed by Dunnett's multiple comparisons test (****$p < 0.0001$). (**b**) qPCR for *KILR* or *KCTD1-5* expression in breast cells after doxycycline induction of ectopic *KCTD1-5* or *KILR* expression. The Tet-CON represents an empty vector control. Error bars, SEM (n = 3). p values were determined by two-way ANOVA followed by Dunnett's multiple comparisons test (****$p < 0.0001$). (**c**) Representative apoptosis analysis of breast cells after doxycycline induction of ectopic *KILR* expression by double staining with annexin V and PI. The Tet-CON represents an empty vector control. The quadrants Q were defined as Q1 = live (Annexin V- and PI-negative), Q2 = early stage of apoptosis (Annexin V-positive/PI-negative), Q3 = late stage of apoptosis (Annexin V- and PI-positive) and Q4 = necrosis (Annexin V-negative/PI-positive). (**d**) The percentage of cells in early- and late-stage apoptosis in each group (Q2 + Q3). Error bars, SEM (n = 3). p values were determined by Student's *t*-test (*$p < 0.05$). (**e**) qPCR for *KILR* expression in cells after doxycycline induction of ectopic *KILR* expression. The Tet-CON represents an empty vector control. Error bars, SEM (n = 3). p values were determined by a Student's *t*-test (**$P < 0.01$).

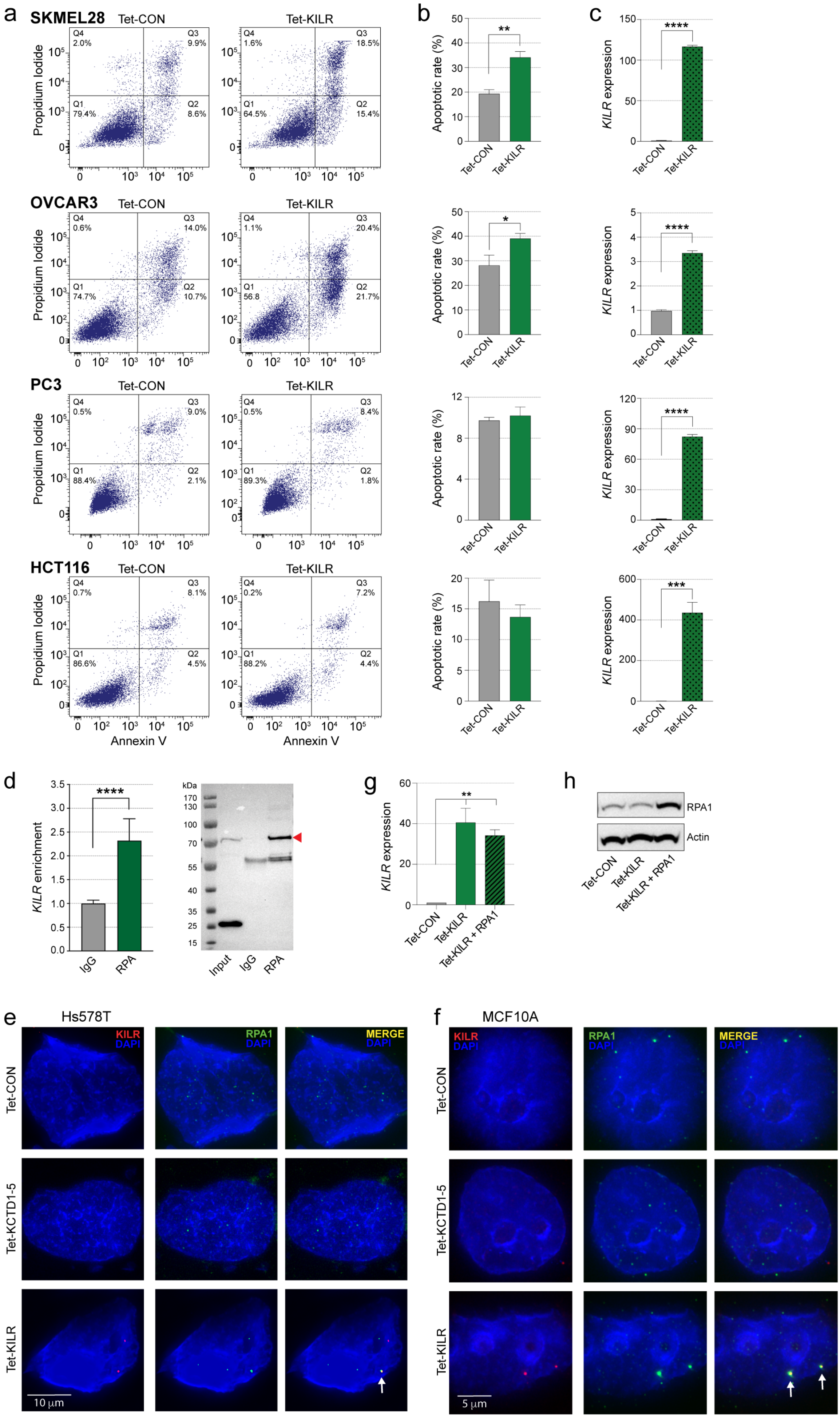

a
SKMEL28
OVCAR3
PC3
HCT116
Tet-CON
Tet-KILR
Propidium Iodide
Annexin V
b
Apoptotic rate (%)
c
KILR expression
d
KILR enrichment
IgG
RPA
Input
kDa
g
Tet-KILR + RPA1
h
RPA1
Actin
e
Hs578T
f
MCF10A
KILR
DAPI
MERGE
Tet-KCTD1-5
10 μm
5 μm

Supplementary Figure 4. *KILR* overexpression induces apoptosis of non-breast cancer cells through sequestration the RPA1 protein. (**a**) Representative apoptosis analysis of skin (SKMEL28), ovarian (OVCAR3), prostate (PC3) and colorectal (HCT116) cancer cells after doxycycline induction of ectopic *KILR* expression by double staining with annexin V and PI. The Tet-CON represents an empty vector control. The quadrants Q were defined as Q1 = live (Annexin V- and PI-negative), Q2 = early stage of apoptosis (Annexin V-positive/PI-negative), Q3 = late stage of apoptosis (Annexin V- and PI-positive) and Q4 = necrosis (Annexin V-negative/PI-positive). (**b**) The percentage of cells in early- and late-stage apoptosis in each group (Q2 + Q3). Error bars, SEM (n = 3). p values were determined by Student's *t*-test (*p < 0.05, **p < 0.01). (**c**) qPCR for *KILR* expression in cells after doxycycline induction of ectopic *KILR* expression. The Tet-CON represents an empty vector control. Error bars, SEM (n = 3). p values were determined by a Student's *t*-test (***P < 0.001, ****p < 0.0001). (**d**) RIP for the binding of *KILR* to RPA1 from MCF7 cells. Left panel: Copurified RNA from RPA1 IPs assayed for *KILR* enrichment by qPCR. Error bars, SEM (n = 3). p values were determined by a Student's *t*-test (****p < 0.0001). Right panel: IP specificity was controlled by RPA1 Western blotting. The red arrowhead denotes full-length RPA1. (**e, f**) Representative confocal microscopy images of *KILR* and RPA1 in Hs578T (**e**) and MCF10A (**f**) cells after doxycycline induction of ectopic *KCTD1-5* or *KILR* expression stained with Stellaris *KILR* RNA FISH probes (red) and immunostained with anti-RPA1 (green) (n = 3). The Tet-CON represents an empty vector control. Nuclei were stained with DAPI (blue). White arrows highlight *KILR*/RPA1 co-localization. Scale bars, 5 or 10 μm. (**g**) qPCR for *KILR* expression in MCF7 cells after doxycycline induction of ectopic *KILR* expression with or without RPA1 overexpression. The Tet-CON represents an empty vector control. Error bars, SEM (n = 3). p values were determined by one-way ANOVA followed by Dunnett's multiple comparisons test (**p < 0.01). (**h**) Western blot of MCF7 cells after doxycycline induction of ectopic *KILR* expression with or without RPA1 overexpression. The Tet-CON represents an empty vector control. Actin was used as a loading control.

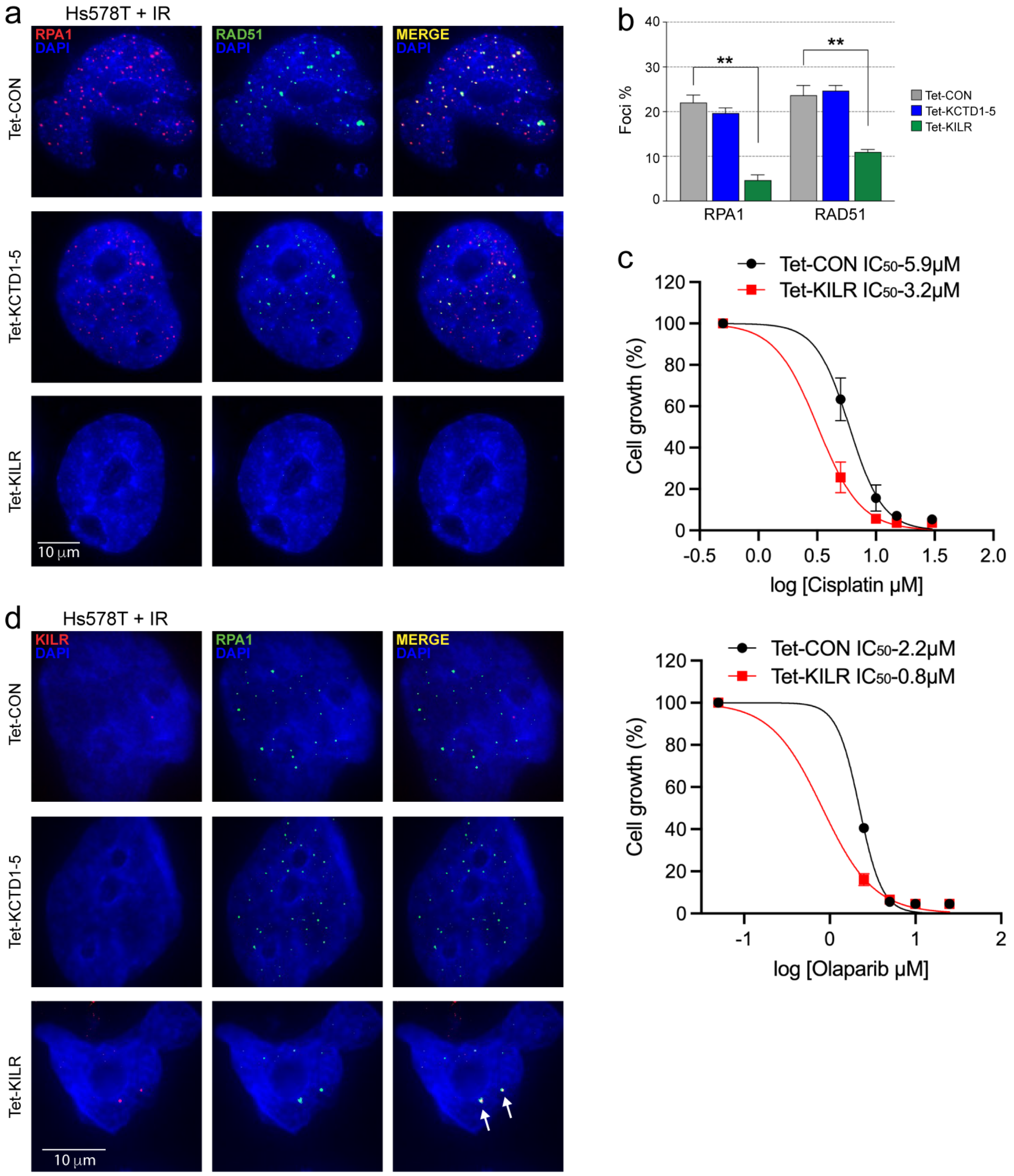

Supplementary Figure 5. *KILR* overexpression inhibits DNA repair and sensitizes breast cancer cells to chemotherapy. (**a**) Representative confocal microscopy images of RPA1 and RAD51 in Hs578T cells after doxycycline induction of ectopic *KCTD1-5* or *KILR* expression and exposure to 6-Gy IR (n = 3). 6 h post-IR, cells were immunostained with anti-RPA1 (red) and anti-RAD51 (green). The Tet-CON represents an empty vector control. Nuclei were stained with DAPI (blue). Scale bar, 10 μm. (**b**) Quantification of RPA1 or RAD51 foci in Hs578T cells. A cell with > 5 distinct RPA1 or RAD51 foci in the nucleus was considered as positive. Error bars, SEM (n = 3). p values were determined by one-way ANOVA followed by Dunnett's multiple comparisons test (**p < 0.01). (**c**) Growth inhibition curves for MCF7 cells treated with cisplatin (top panel; n = 3) or olaparib (bottom panel; n = 2). (**d**) Representative confocal microscopy images of *KILR* and RPA1 in Hs578T cells after doxycycline induction of ectopic *KCTD1-5* or *KILR* expression and exposure to 6-Gy IR (n = 3). 6 h post-IR, cells were stained with Stellaris *KILR* RNA FISH probes (red) and immunostained with anti-RPA1 (green). The Tet-CON represents an empty vector control. Nuclei were stained with DAPI (blue). White arrows highlight *KILR*/RPA1 co-localization. Scale bar, 10 μm.

Supplementary References

1. Storer, J., Hubley, R., Rosen, J., Wheeler, T.J. & Smit, A.F. The Dfam community resource of transposable element families, sequence models, and genome annotations. Mob DNA 12, 2 (2021).
2. Hou, M. et al. LocExpress: a web server for efficiently estimating expression of novel transcripts. BMC Genomics 17, 1023 (2016).
3. Ke, L., Yang, D.C., Wang, Y., Ding, Y. & Gao, G. AnnoLnc2: the one-stop portal to systematically annotate novel lncRNAs for human and mouse. Nucleic Acids Res 48, W230-W238 (2020).
4. Li, J. et al. TANRIC: An Interactive Open Platform to Explore the Function of lncRNAs in Cancer. Cancer Res 75, 3728-37 (2015).
